# Supplementary material for: High-Resolution Genotyping of Expressed Equine MHC Reveals a Highly Complex MHC Structure
Source: Genes (Basel). 2023 Jul 10;14(7):1422. doi: 10.3390/genes14071422 (PMC10379315; doi:10.3390/genes14071422)
Supplement: Supplementary file 1 [file genes-14-01422-s001.zip › Supplementary Data legends.pdf]

Supplementary Data S1 - Summary of MHCI and MHCII sequencing data for a cohort of Thoroughbred horses. For both the MHCI and MHCII data the sample identification (column A) and the breed of the animal from which the sample was taken (column B - TB = Thoroughbred) are shown **MHCI:** Comments on the MHCI haplotyping are provided in column C; Homozygous = a single MHCI haplotype identified in the sample, Unassigned alleles = alleles which could not be assigned to MHC I haplotypes identified in the animal. The number of reads for each sample that passed filtering and were used for haplotyping from the For1/Rev2.2 and For3.2/Rev1 primer pairs are recorded in columns D and E respectively. The haplotypes identified in each animal are shown in columns F and G. Details for the alleles in haplotype 1 and 2 are shown in columns H-W and Y-AN respectively and details of alleles that couldn't be assigned to a haplotype are shown in columns AP-BI (unassigned alleles for sample 38314M, which are thought to compromise either 1 or 2 haplotypes are shown on a green background). An asterisk is used to identify haplotypes which were incomplete due to absence of alleles which are generally found at low frequency - this was assumed to represent a 'drop-out' where the frequency of alleles was beyond the resolution of the sequencing. **MHCII:** Comments on the MHCII haplotyping are provided in column C; Homozygous = a single MHCII haplotype identified in the sample, Unassigned alleles = alleles which could not be assigned to MHCII haplotypes identified in the animal. The number of reads for each sample that passed filtering and were used for haplotyping for DQA, DQB, DRA and DRB are shown in columns D-G and the defined haplotypes for each individual are shown in columns H and I. Details of the sequences in MHCII haplotype1 and 2 are shown in columns J-Q and S-Z respectively and details of alleles that couldn't be assigned to a haplotype are shown in columns AB-AD.

Supplementary Data S2 - Fasta files for each of the novel MHCI and MHCII alleles identified in this study. The sequence data is for 152 novel MHCI allele sequences and 117 novel MHCII allele sequences.

Supplementary Data S3 - Summary of MHCI and MHCII sequencing data for a cohort of Icelandic and Norwegian Fjord Horses. For both the MHCI and MHCII data the sample identification (column A) and the breed of the animal from which the sample was taken (column B) are shown **MHCI:** Comments on the MHCI haplotyping are provided in column C; Homozygous = a single MHCI haplotype identified in the sample, Unassigned alleles = alleles which could not be assigned to MHCI haplotypes identified in the animal. The number of reads for each sample that passed filtering and were used for haplotyping from the For1/Rev2.2 and For3.2/Rev1 primer pairs are recorded in columns D and E respectively. The haplotypes identified in each animal are shown in columns F and G. Details for the alleles in haplotype 1 and 2 are shown in columns H-P and R-Z respectively and details of alleles that couldn't be assigned to a haplotype are shown in columns AB-AL (unassigned alleles for sample Norw_037, which are thought to compromise either 1 or 2 haplotypes are shown on a green background). Universal alleles (excluding alleles for Eqca-2*001 which are included within the haplotypes) are shown in columns AN-BB. An asterisk is used to identify haplotypes which were incomplete due to absence of alleles which are generally found at low frequency - this was assumed to represent a 'drop-out' where the frequency of alleles was beyond the resolution of the sequencing. **MHCII:** Comments on the MHCII haplotyping are provided in column C; Homozygous = a single MHCII haplotype identified in the sample, Unassigned alleles = alleles which could not be assigned to MHCII haplotypes identified in the animal, DRB anomaly = haplotypes in which the repertoire of DRB alleles appeared to be anomalous (no DRB1 identified and many DRB alleles unassigned). The number of reads for each sample that passed filtering and were used for haplotyping for DQA, DQB, DRA and DRB are shown in columns D-G and the defined haplotypes for each individual are shown in columns H and I. Details of the sequences in MHCII haplotype 1 and 2 are shown in columns J-S and U-AD respectively and details of alleles that couldn't be assigned to a

haplotype are shown in columns AF-AI. Details of the unassigned DRB alleles in individuals bearing the anomalous DRB repertoires are shown in columns AK-AV; there were some repetitive patterns of co-segregating alleles which are shown in coloured groups.
